# Rethinking sensorimotor circuits

**DOI:** 10.7554/eLife.104111

**Published:** 2024-11-13

**Authors:** Maarten F Zwart

**Affiliations:** 1 School of Psychology and Neuroscience and Centre of Biophotonics, University of St Andrews St Andrews United Kingdom

**Keywords:** vestibular, development, sensorimotor, neural circuits, Zebrafish

## Abstract

New research shows that the neural circuit responsible for stabilising gaze can develop in the absence of motor neurons, contrary to a long-standing model in the field.

**Related research article** Goldblatt D, Rosti B, Hamling KR, Leary P, Panchal H, Li M, Gelnaw H, Huang S, Quainoo C, Schoppik D. 2024. Motor neurons are dispensable for the assembly of a sensorimotor circuit for gaze stabilization. *eLife* **13**:RP96893. doi: 10.7554/eLife.96893.

As you nod in agreement, or furiously shake your head side-to-side, your gaze stays almost perfectly stable. This remarkable ability to fix your eyes on whoever you may be agreeing (or disagreeing) with relies on a sensorimotor neural circuit in the brain that adjusts eye position to accommodate shifts in head orientation.

Sensorimotor circuits are made up of three key components: (i) sensory neurons that detect external stimuli; (ii) projection neurons that transmit information from sensory neurons; and (iii) motor neurons that receive this information and innervate muscle cells to produce a response. In the circuit that stabilizes gaze – known as the vestibulo-ocular reflex (or VOR for short) – the sensory neurons detect changes in balance caused by movements of the head (Figure 1A). This information is then relayed via the projection neurons to extraocular motor neurons, which move the eyes in the opposite direction of the movement of the head in order to maintain visual stability.

**Figure 1.**
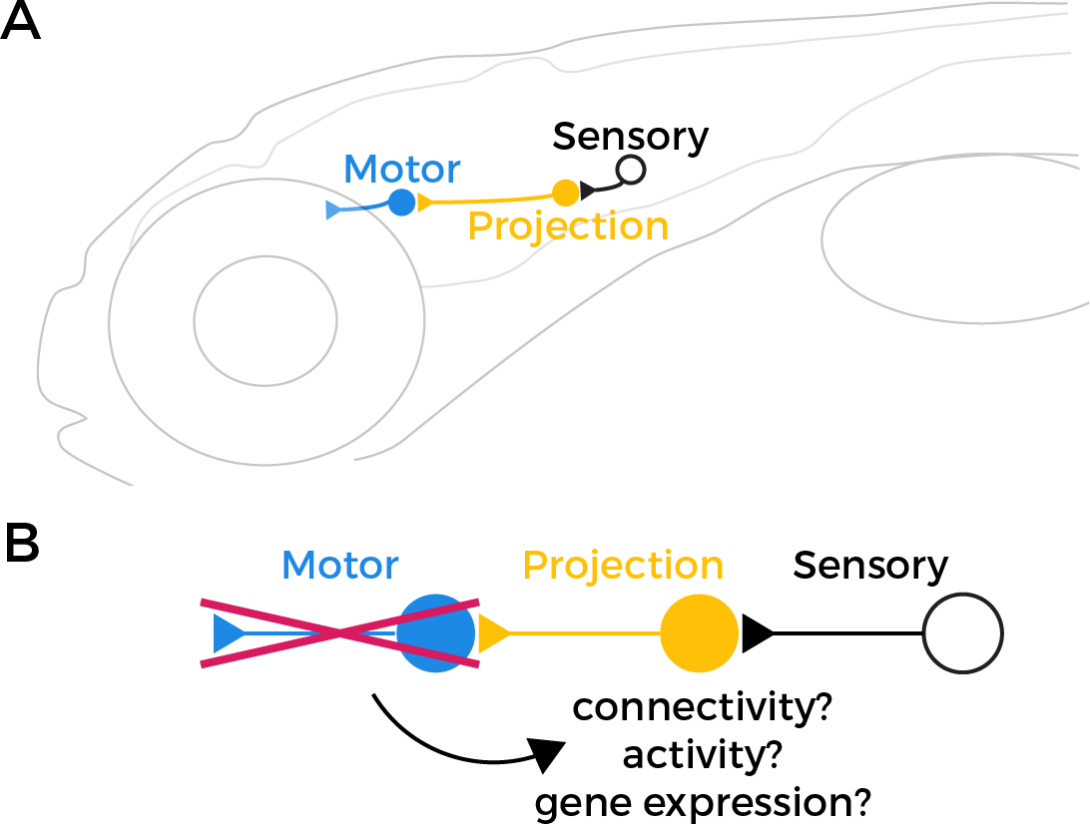
Testing the retrograde specification model. (**A**) The vestibulo-ocular reflex (VOR) circuit in zebrafish larvae contains three core components: sensory neurons (black), projection neurons (yellow), and extraocular motor neurons (blue). The sensory neurons detect changes in head orientation and relay this information via synapses (represented as triangles) to the projection neurons, which then pass it to the motor neurons that innervate muscles in the eye. (**B**) It has been proposed that motor neurons have an important role in specifying the fate of projection neurons during development. However, when Goldblatt et al. tested this ‘retrograde specification model’ by genetically removing motor neurons from the VOR circuit in zebrafish larvae, they found that the mutations did not alter the connectivity, activity or gene expression of the projection neurons.

Graphic of zebrafish in panel A adapted from Yang et al., 2022.

For decades, scientists have theorised that motor neurons have an essential role during the development of sensorimotor circuits that involves specifying the fate of the projection neurons (Glover, 2000; Glover, 2003). This ‘retrograde specification model’ was in part inspired by the observation that extraocular motor neurons establish connections with muscle cells before projection neurons connect to them. This suggests that projection neurons might require additional cues to know what sensory information to transmit and which cells to target. Now, in eLife, David Schoppik and colleagues at New York University – including Dena Goldblatt as first author – report new findings that challenge this theory (Goldblatt et al., 2024).

To test the retrograde specification model, the team studied the development of the VOR circuit in zebrafish larvae – a model organism that is ideal for investigating the nervous system due to its transparency, relatively simple neural circuitry and genetics. Goldblatt et al. removed a gene called *phox2a* that specifies the fate of extraocular motor neurons to prevent the zebrafish larvae from developing these types of cells (Guo et al., 1999; Nakano et al., 2001; Coppola et al., 2005; Hasan et al., 2010).

Before testing whether this modification affects the wiring of the VOR circuit, Goldblatt et al. confirmed that the *phox2a* mutant larvae were definitely lacking extraocular motor neurons. These neurons typically express the transcription factor *isl1* and are located in cranial nucleus III and cranial nucleus IV, two regions in the brain that control eye movement (Greaney et al., 2017). Goldblatt et al. found the mutation nearly eliminated all *isl1-*expressing cells. They were also unable to detect another motor neuron marker called *vachta*. Moreover, the eyes of the mutant larvae rotated toward their ears, an effect that is often seen when motor neurons are lost from cranial nuclei III and IV.

According to the retrograde specification model, projection neurons in the VOR circuit in the mutant zebrafish should no longer be able to receive sensory information about how the head is tilted, because they either failed to develop or are mis-wired. However, Goldblatt et al. found that projection neurons in the *phox2a* mutants maintained normal patterns of activity, and their anatomy and quantity was unaffected. This suggests that the connections between sensory and projection neurons in the VOR circuit can still form correctly even in the absence of functional motor neurons (Figure 1B).

Further experiments revealed that removal of *phox2a* did not cause the axons of the projection neurons to misroute or connect to the wrong target. In addition, the set of genes that assemble the synapses of projection neurons in wildtype cells were still expressed in the mutant larvae. This suggests that the projection neurons do not require extraocular motor neurons to select and form connections with the correct target cells.

Finally, Goldblatt et al. tested if the projection neurons depend on their target motor neurons to determine which genes to express. They found no meaningful differences in gene expression between the *phox2a* mutants and wild-type larvae, providing further evidence that projection neurons can develop normally without their pre-synaptic motor neuron partners.

These findings are significant not only for understanding the VOR, but also for other sensorimotor circuits. Similar principles might apply to circuits in the spinal cord that control motor responses to other types of sensory stimuli. The study also adds to a growing body of evidence suggesting that certain neurons may develop more independently than previously thought (Sürmeli et al., 2011; Sweeney et al., 2018), an insight that could reshape our understanding of how sensorimotor circuits form across the nervous system.
